# Combining Organophosphate Treated Wall Linings and Long-lasting Insecticidal Nets for Improved Control of Pyrethroid Resistant *Anopheles gambiae*

**DOI:** 10.1371/journal.pone.0083897

**Published:** 2014-01-07

**Authors:** Corine Ngufor, Emile Tchicaya, Benjamin Koudou, Sagnon N'Fale, Roch Dabire, Paul Johnson, Hilary Ranson, Mark Rowland

**Affiliations:** 1 London School of Hygiene and Tropical Medicine, London, United Kingdom; 2 Centre Suisse de Recherche Scientifiques, Abidjan, Cote D'Ivoire; 3 Centre National de Recherche et de Formation sur le Paludisme, Ouagadougou, Burkina Faso; 4 Centre Muraz, Bobo-Dioulasso, Burkina Faso; 5 Robertson Centre for Biostatistics, University of Glasgow, Glasgow, United Kingdom; 6 Liverpool School of Tropical Medicine, Liverpool, United Kingdom; University of Crete, Greece

## Abstract

**Background:**

New approaches to delivering insecticides need to be developed to improve malaria vector control. Insecticidal durable wall lining (DL) and net wall hangings (NWH) are novel alternatives to indoor residual spraying which can be produced in a long-lasting format. Non-pyrethroid versions could be used in combination with long-lasting insecticidal nets for improved control and management of insecticide resistant vector populations.

**Methods:**

Experimental hut trials were carried out in Valley du Kou, Burkina Faso to evaluate the efficacy of pirimiphos methyl treated DL and NWH either alone or in combination with LLINs against pyrethroid resistant *Anopheles gambiae ss*. Comparison was made with pyrethroid DL. Mosquitoes were genotyped for *kdr* and *ace-1R* resistant genes to investigate the insecticide resistance management potential of the combination.

**Results:**

The overall *kdr* and *ace-1^R^* allele frequencies were 0.95 and 0.01 respectively. Mortality with p-methyl DL and NWH alone was higher than with pyrethroid DL alone (>95% vs 40%; P<0.001). Combining pyrethroid DL with LLINs did not show improvement in mortality (48%) compared to the LLIN alone (44%) (P>0.1). Combining p-methyl DL or NWH with LLINs reduced biting rates significantly (8–9%) compared to p-methyl DL and NWH alone (>40%) and killed all *An gambiae* that entered the huts. Mosquitoes bearing the *ace-1^R^* gene were more likely to survive in huts with p-methyl DL alone (p<0.03) whereas all resistant and susceptible genotypes were killed by the combination.

**Conclusion:**

P-methyl DL and NWH outperformed pyrethroid DL. Combining p-methyl DL and NWH with LLINs could provide significant epidemiological benefits against a vector population which is resistant to pyrethroids but susceptible to organophosphates. There was evidence that the single intervention would select *kdr* and *ace-1^R^* resistance genes and the combination intervention might select less strongly. Technology to bind organophosphates to plastic wall lining would be worth developing.

## Introduction

Malaria vector control largely depends on a limited collection of tools. Long lasting insecticidal nets (LLINs) and indoor residual spraying (IRS) have contributed significantly to the recent reductions in malaria morbidity and mortality burdens [1], and these interventions are reliable and effective in a wide range of situations. LLINs are easy to deliver even in the most remote communities and hence have been more widely deployed in malaria endemic countries in sub-Saharan Africa. IRS requires more complex operational delivery systems; it is thus mostly used in a targeted approach. Alternative efficacious and practical tools for delivering insecticides indoors need to be urgently developed in order to diversify the “tool-box” for malaria vector control and to enhance capacity to effectively interrupt malaria transmission in holo-endemic areas in sub-Saharan Africa.

The covering of home walls with insecticidal materials is a novel approach that simulates IRS. Insecticide treated plastic wall linings also known as durable lining (DL) can be produced via the long-lasting net technology which incorporates the insecticide into the fibres before yarn extrusion. Long-lasting pyrethroid DL when used on interior walls, showed high acceptability and little or no decline in bioefficacy after 12–15 months with minimal loss of insecticide [2], [3]. Due to the long-lasting technology, it is hoped that pyrethroid DL may only need to be replaced on walls after 3–4 years. It can therefore be regarded as a long-lasting alternative to IRS which would be vital for high malaria transmission areas where recurrent IRS treatments are normally required for interruption of transmission. DL also has the advantage of providing a more uniform covering of the wall with insecticide compared to IRS and of improving the interior appearance of traditional dwellings especially in rural areas [3]. However, in the current era of pyrethroid resistance [4] [5], the future of pyrethroid DL is rather questionable. Studies on pyrethroid DL in West Africa revealed relatively low mortality rates of 37–47% against pyrethroid resistant mosquitoes in experimental hut trials [6] [7]. Mortality rates >70% have been recorded with pyrethroid IRS in a pyrethroid susceptible area in West Africa [8]. To reduce selection pressure for pyrethroid resistance on malaria vectors, the WHO recommends that pyrethroids should be reserved only for treating LLINs since they remain the most appropriate class of insecticides for this purpose [9]. This requires that DL treated with alternative insecticides should be urgently investigated and developed. One potential candidate insecticide is the WHO-approved organophosphate insecticide pirimiphos methyl (p-methyl). A new micro-encapsulated formulation of p-methyl (Actellic CS) shows residual activity for up to 9 months as an IRS treatment on cement walls and has been shown to control pyrethroid resistant *An gambiae* [10].

In rural Africa, householders often cover their walls with wall hangings made from netting material to improve interior aesthetic appearance. Insecticide treated net wall hangings (NWH) could function in a manner which is similar to DL and could be a more acceptable, practical and innovative means for delivering insecticides indoors. Curtains treated with pyrethroids have been shown to be effective against vectors of dengue in South America [11] [12]. The potential of such materials to control malaria vectors is yet to be fully explored.

It is now clear that the development and rapid spread of insecticide resistance in *An gambiae* populations across Africa [4] [13] is well capable of undermining vector control [8] [14]–[16]. The World Health Organisation (WHO) calls for an immediate pro-active response to insecticide resistance to sustain the effectiveness of malaria vector control [9] [17]. This requires investigating ways in which insecticide resistance management can be applied for vector control. One available strategy is to combine interventions which deliver unrelated insecticides in the same place and at the same time [17]. This approach has potential to improve the control of the insect vector population and manage the spread of insecticide resistant insect genotypes [18] [19]. The latter is based on the concept that insect genotypes which are resistant to the insecticide in one intervention can be killed by the insecticide in the other intervention [20].

The aim of the current study was to investigate via a series of experimental hut trials whether DL or NWH treated with pirimiphos methyl (p-methyl) CS applied alone or in combination with LLINs has the potential to control malaria transmitted by pyrethroid resistant *Anopheles gambiae* s.s. in Burkina Faso. Comparison was made to currently available pyrethroid DL. Using molecular genotyping studies, the capacity of the combination to potentially manage insecticide resistance by preventing the selection of organophosphate and pyrethroid resistant genotypes was also investigated.

## Materials and Methods

### Experimental huts

The trials were carried out at the Centre Muraz experimental hut station in Valley du Kou 5 (4°25′W, 11°24′N) situated near Bobo-Dioulasso, in South-western Burkina Faso. The station is surrounded by a huge rice growing valley. The rainy season extends from June to October and the dry season from November to May. The rice paddies provide extensive breeding sites for mosquitoes throughout the year. The two molecular forms M and S of *An gambiae s.s.* occur in sympatry notably at the end of the rainy season [21]. The study was performed in 6 experimental huts of the WHOPES approved West African design between August and November of 2011. Permission to use the hut station was obtained from Centre Muraz. The experimental huts are built on concrete plinths surrounded by water-filled moats to prevent entry of scavenging ants. Veranda traps capture the exiting mosquitoes. The huts are made of brick plastered with cement on the inside, with a corrugated iron roof. The ceiling is made of thick polyethylene sheeting and the walls have four window slits (1 cm gap) through which mosquitoes enter. Prior to the study, huts were refurbished to reduce any possibility of contamination from previous trials.

### Susceptibility tests

During the trials, samples of adult *An. gambiae* which emerged from larvae collected from the experimental hut site (Valley du Kou 5) were tested in WHO test kits for susceptibility to pyrethroids using deltamethrin 0.05% treated papers and to organophosphates using p-methyl 0.25% treated papers. 0.25% was used as a diagnostic dose for p-methyl based on preliminary studies which showed a concentration of ∼0.1% induced 100% mortality in the *An gambiae* kisumu laboratory susceptible strain (Ranson et al, unpublished data).

### Experimental hut treatments

Three experimental hut trials were carried out. The first two trials lasted 6 weeks and the third lasted 4 weeks. The first trial aimed to evaluate the efficacy of p-methyl treated DL and NWH against pyrethroid resistant *An gambiae*, comparing them to currently available pyrethroid DL (ZeroVector®, Vestergaard Frandsen, Switzerland). The level of interior coverage required for optimum impact (walls only versus walls and ceiling) was also investigated. The following six single treatments were tested in the first trial:

1. Untreated Control (untreated plastic sheeting)
2. Pyrethroid treated durable lining (ZeroVector®, Vestergaard Frandsen, Switzerland) on walls
3. P-methyl CS treated durable lining (p-methyl DL) on walls
4. P-methyl CS treated net wall hangings (p-methyl NWH) on walls
5. P-methyl DL on walls and ceilings
6. P-methyl NWH on walls and ceilings

In the second experimental hut trial, the p-methyl DL and NWH were combined with LLINs and compared to LLINs alone and p-methyl DL and NWH alone. The following six interventions were tested:

1. Untreated Net with 6 holes
2. Pyrethroid LLIN (Permanet® 2.0 Vestergaard Frandsen, Switzerland), with 6 holes
3. P-methyl DL on walls and ceilings
4. P-methyl NWH on walls and ceilings
5. P-methyl DL on walls and ceilings+Pyrethroid LLIN with 6 holes
6. P-methyl NWH on walls and ceilings+Pyrethroid LLIN with 6 holes

In the third trial we compared the combination of pyrethroid DL and LLIN to the combination of p-methyl DL and LLINs. The aim of this trial was to explore the advantage of p-methyl DL over currently available pyrethroid DL to see whether there was any benefit to using the organonophosphate over the pyrethroid on the lining material in a situation of high pyrethroid resistance frequency. The following treatments were tested:

1. Untreated Net with 6 holes
2. Pyrethroid LLIN (Permanet® 2.0 Vestergaard Frandsen, Switzerland), with 6 holes
3. Pyrethroid DL (ZeroVector®, Vestergaard Frandsen, Switzerland)on walls and ceilings+Pyrethroid LLIN with 6 holes
4. P-methyl NWH on walls and ceilings+Pyrethroid LLIN with 6 holes

### Treatment of materials

The DL was 50% shade cloth made of woven high density polyethylene (HDPE) plastic (Capatex Ltd, UK). The NWH was 100 denier nylon netted fabric purchased from the local market. These materials were treated at 1 g/m^2^ with micro-encapsualted primiphos methyl (p-methyl) CS (Actellic® 300CS [PP511 CS]) provided by Syngenta. The insecticide was applied onto the plastic sheets by spraying with a Hudson Xpert knapsack sprayer and to nettings by hand dipping. Treated materials were left to dry for 24 hours in the shade before being set up in the experimental huts. Pyrethroid treated DL used in the study was HDPE woven fibre sheet factory treated with deltamethrin at 175 mg/m^2^. The LLIN (Permanet® 2.0, Vestergaard Frandsen, Switzerland) was WHOPES approved, made of polyester fibres, factory-coated with a wash- resistant formulation of deltamethrin at a target dose of 55 mg/m^2^. To simulate wear and tear, bednets were intentionally holed with six 16 cm^2^ diameter holes (4 at the sides and 2 at the ends) according to WHOPES guidelines.

### Setting up treated materials to walls

In order to minimise contamination of the hut walls when rotating the treatments, a removable underlying layer of untreated material (plastic lining) was used to separate the walls from the treated materials and these were rotated along with the respective treatments. Treated plastic sheeting were pinned to small battens that had been nailed unto the walls while treated netting were hung unto nails fitted at the edges of the ceiling. These methods of fixing the treated materials unto the walls also allowed the treatments to be easily rotated between huts on a weekly basis.

### Rotation of sleepers and treatments

Treatments were allocated to the six experimental huts and rotated each week using a randomised Latin square design to adjust for any differential positional attractiveness of the huts. Weekly rotation with one day for cleaning between rotations minimised any carry over effect between the treatments. Six adult men served as volunteer sleepers to attract mosquitoes into the huts. They were rotated between huts on successive nights to adjust for any variation in individual attractiveness to mosquitoes. They slept in the huts from 20:00 to 05:00 each night. Mosquitoes were collected each morning at 05:00 from under bed nets, floors, walls, ceilings and verandas using collection tubes and torches. The collections were transported to the laboratory where mosquitoes were identified to species and scored as blood fed or unfed and live or dead. Live mosquitoes were held in netted plastic cups and supplied with 10% glucose solution and delayed mortality was recorded after 24 h. Male mosquitoes were not scored.

### Entomological Outcomes

The entomological impact of each treatment in this study was expressed in terms of the following entomological outcomes;

1. Deterrence: percentage reduction in the number of mosquitoes caught in treated hut relative to the number caught in the control hut
2. Exiting rates due to potential irritant effect of treatments expressed as percentage of the mosquitoes collected from the veranda trap
3. Inhibition of blood feeding: reduction in blood feeding rate relative to the control. This was as follows:
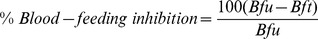
Where Bfu is the proportion of blood-fed mosquitoes in the untreated control huts and Bft is the proportion of blood-fed mosquitoes in the huts with a specific insecticide treatment;
4. Mortality: percentage of dead mosquitoes in treated hut at the time of collection and after a 24 h holding period corrected for control mortality.
5. The personal protective effect of the treatments which is described by a reduction in the number of blood-fed mosquitoes relative to the control hut was calculated as follows:
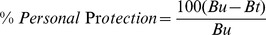
Where Bu = is the number of blood-fed mosquitoes in the untreated control huts and Bt is the number of blood-fed mosquitoes in the huts with insecticide treatments.
6. The overall insecticidal effect of a treatment relative to the number of mosquitoes that would ordinarily enter an untreated control hut was estimated by using the following formula and expressed as a percentage:
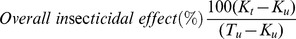
where K_t_ is the number killed in the treated hut, K_u_ is the number dying in the untreated control hut, and T_u_ is the total number collected from the control hut.

### Residual activity of insecticide treatments

To measure residual activity, WHO cone bioassays were undertaken on treated materials in situ using the laboratory-susceptible *An. gambiae s.s.* Kisumu strain. Adult females 3–5 days old were introduced into cones fixed to treated plastic sheeting/net wall hangings (for 30 minutes) and LLINs (for 3 minutes) according to WHO guidelines [22]. For each trial, approximately 40–50 adult females were tested in batches of 10 mosquitoes on each type of treated material each week. These were held in netted plastic cups, provided 10% glucose solution and mortality recorded after 24 hours.

### Studies on selection of insecticide resistance genes

Samples of *An gambiae* (dead and alive) collected from the respective experimental hut treatments through the course of the trials were preserved for molecular analysis. Only samples from the first and second experimental hut trial were analysed. These mosquitoes were systematically selected from the alive and dead collections of the first trial to cover the entire period of the trial and to include equal numbers of bloodfed and unfed mosquitoes. For the second trial, because the numbers entering the huts had reduced, we analysed all samples collected. Genomic DNA was extracted using the Livak procedure [23]. Samples were identified to species and molecular form of *An gambiae* using SINE-PCR. Molecular detection of the *kdr* (L1014F) and *ace-1^R^* (G119S) mutation alleles was carried out by real-time Taqman PCR as described by Bass et al [24].

### Statistical analysis

The effects of the different experimental hut treatments on each of the main entomological outcomes (bloodfeeding, exophily and mortality) were assessed using binomial generalised linear mixed models (GLMMs) with a logit link function, fitted using the ‘lme4’ package for R. A separate model was fitted for each outcome. In addition to the fixed effect of each treatment, each model included random effects to account for the following sources of variation: between the 6 huts used in the studies; between the 6 sleepers who slept in the huts; between the 6 weeks of the trial; and finally an observation-level random effect to account for variation not explained by the other terms in the model (overdispersion). In comparing fixed effects between treatments, the binomial GLMM cannot estimate mortalities of exactly 0 or 100%, because the logits of 0 and 1 are undefined. Some treatments caused 100% mortality during the second hut trial; hence it was not possible to fit a valid GLMM to this data. To overcome this problem, a small constant (0.5) was added to rows contributing to zero cell counts in this data before modelling the GLMM, allowing conservative estimates of treatment effects and p-values to be derived [25]. The numbers entering the huts were analysed using negative binomial regression.

For genotyping data, differences in survival of resistant genotypes for each treatment was analysed by Chi square and Fisher's exact test. All analyses were performed using R version 2.12.2 for Windows [26].

### Ethics Statement

Ethical approval for the study was obtained from the Ethics Review Board of the London School of Hygiene and Tropical Medicine (Approval No. 5872) and from the ‘Comite d'Ethique pour la Recherches en Sante’ (Approval No. 2011-6-33) of the Ministry of Scientific Research of Burkina Faso. Permission to use the experimental hut station was obtained from Centre Muraz. Written informed consent was obtained from the volunteers who slept in the experimental huts to attract mosquitoes.

## Results

### Susceptibility tests

*An gambiae* from Valley du Kou 5 was very resistant to pyrethroids recording 2% mortality with deltamethrin (0.5%) treated papers (Table 1). In contrast, mortality with pirimiphos methyl (0.25%) treated papers was 100% showing that the vector population was largely susceptible to the organophosphate (Table 2).

**Table 1:** Susceptibility of wild *An gambiae ss* from Valley du Kou 5 (VK5) to deltamethrin (0.05%) in WHO cylinder bioassays.

Species	No. tested	% KD (95% CI)	24 h % mortality (95% CI)
*An gambiae s.s.* (Kisumu)	100	100 (96–100)	100 (96–100)
*An gambiae s.s.* (VK5)	100	5 (0–12)	2 (0–7)

**Table 2:** Susceptibilty of wild *An gambiae ss* from Valley du Kou 5 (VK5) to pirimiphos methyl (0.25%) in WHO cylinder bioassays.

Species	No. tested	% KD (95% CI)	24 h % mortality (95% CI)
*An gambiae s.s.* (Kisumu)	100	87 (80–94)	100 (96–100)
*An gambiae s.s.* (VK5)	102	86 (79–92)	100 (96–100)

### Experimental hut trials

Over 5000 *An gambiae* ss were collected from the experimental huts during the trials. The numbers of *Culex quinquefasciatus* collected were too few to permit further analysis.

**Figure 1:**
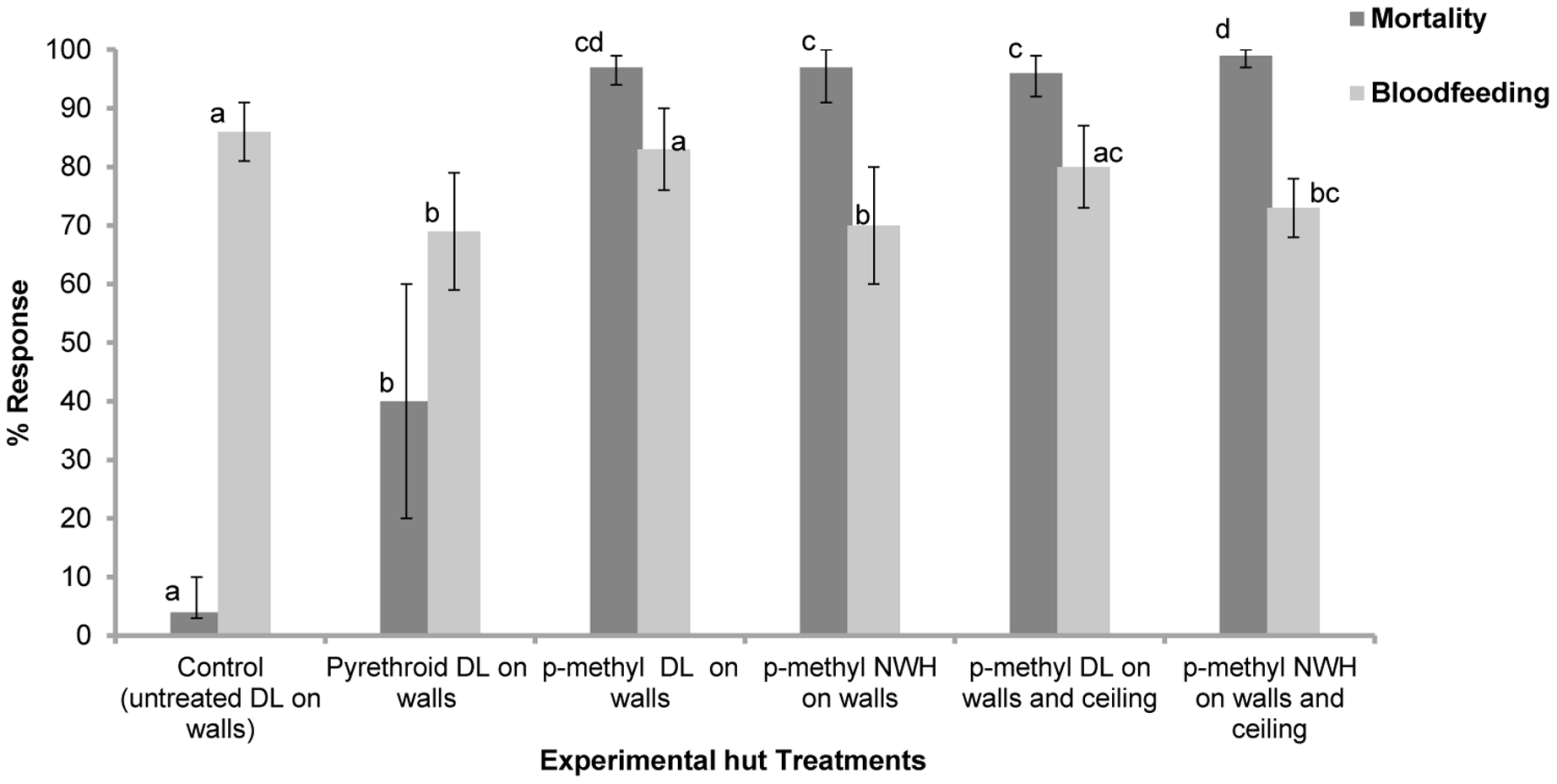
Mortality and bloodfeeding rates of pyrethroid resistant *An gambiae* in experimental huts with single interventions. Percentage mortality (dark shade) and bloodfeeding (lighter shade) of pyrethroid resistant *An gambiae* in experimental huts in Valley du Kou with the indicated single treatments. P-methyl DL and NWH are compared to pyrethroid DL and an untreated control. For each response parameter (mortality or bloodfeeding), values for histograms sharing the same letter label are not significantly different (P>0.05).

**Figure 2:**
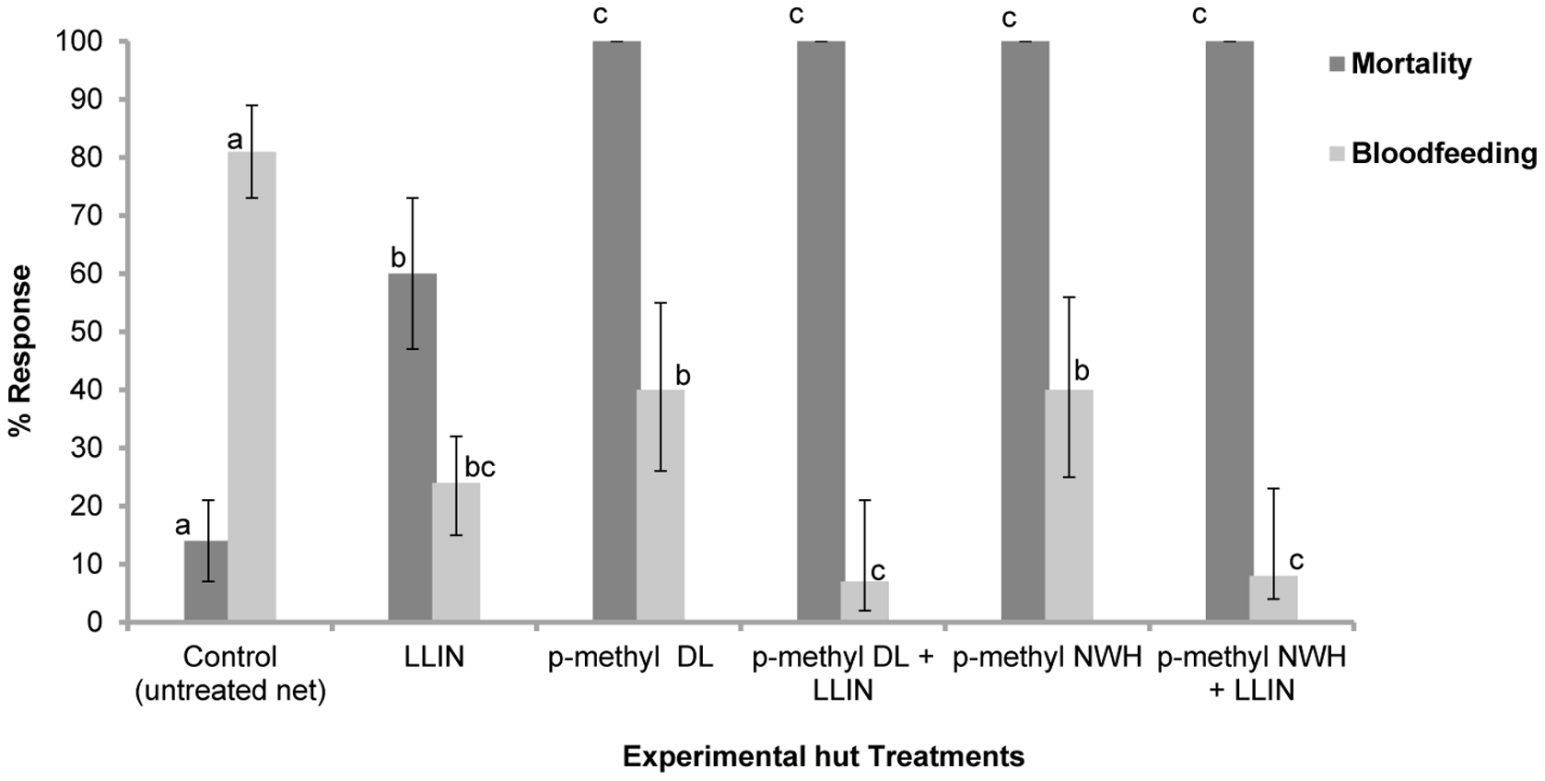
Mortality and bloodfeeding rates of pyrethroid resistant *An gambiae* in experimental huts with combined interventions. Percentage mortality (dark shade) and bloodfeeding (lighter shade) of pyrethroid resistant *An gambiae* in experimental huts in Valley du Kou with the combined p-methyl wall treatment+LLINs versus single treatments alone. For each response parameter (mortality or bloodfeeding), values for histograms sharing the same letter label are not significantly different (P>0.05).

**Figure 3:**
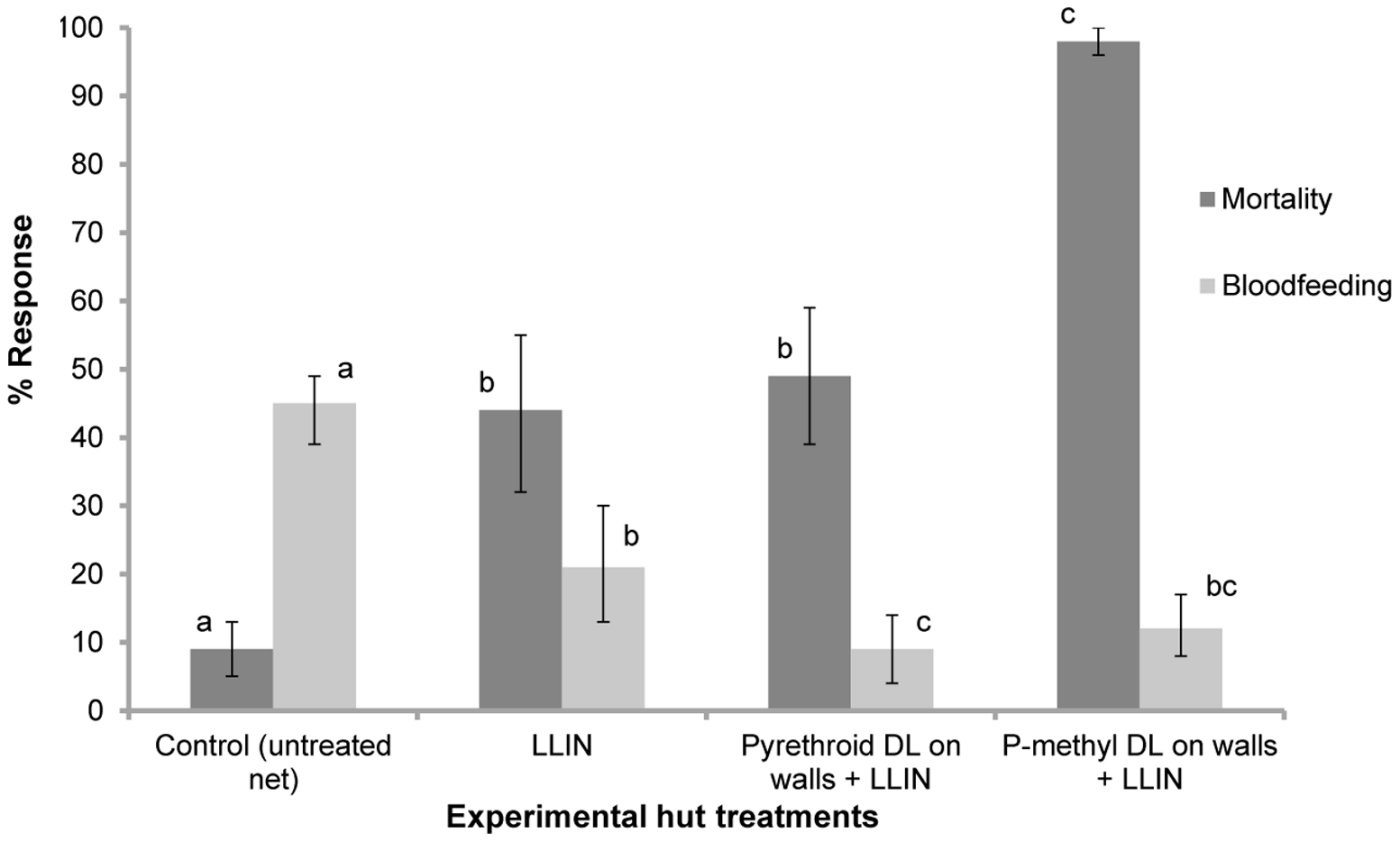
Mortality and bloodfeeding rates of pyrethroid resistant *An gambiae* in experimental huts (third trial). Percentage mortality (dark shade) and bloodfeeding (lighter shade) of pyrethroid resistant *An gambiae* in experimental huts in Valley du Kou with combination of p-methyl DL and LLIN versus combination of pyrethroid DL+LLIN. For each response parameter (mortality or bloodfeeding), values for histograms sharing the same letter label are not significantly different (P>0.05).

1. **Single intervention trial:** A total of 3933 *An gambiae* ss were collected from the experimental huts during the trial. The results obtained are presented in Table 3 and Figure 1. Blood-feeding rates were generally high (70–83%) with the DL and NWH alone (Figure 1) since mosquitoes would normally feed on the person sleeping in the hut before resting on the wall. Hence the treatments provided limited blood-feeding inhibition (4–20%) and personal protection (29–56%) (Table 3). Mortality with pyrethroid DL was 40% (Figure 1). P-methyl treated DL and NWH induced much higher mortality rates (>95%) than pyrethroid DL (P<0.001). With only walls covered, p-methyl DL and NWH showed a similar performance. Highest mortality was attained when all hut surfaces (walls and ceiling) were covered with p-methyl NWH (99%) and hence for the follow on trials, the p-methyl DL and NWH treatments tested were applied on walls and ceiling.
2. **First combined intervention trial:** A total of 320 *An gambiae* ss were collected from the experimental huts during the second trial, far fewer than in the first trial. By this time, the rice in the fields had grown significantly and covered the breeding sites leading to lower numbers of mosquitoes entering the huts compared to the first trial. The results obtained in this trial are presented in Table 4 and Figure 2. The holed LLIN was more protective (23% bloodfed and 0% found inside the net) than the untreated holed net (81% bloodfed and 36% found inside the net) (Table 4 and Figure 2). Combining p-methyl DL and NWH with LLINs reduced bloodfeeding rates significantly (8–9% bloodfed) compared to p-methyl DL and NWH alone (Figure 2) (P<0.001). The combination therefore provided more bloodfeeding inhibition (90–91%) and personal protection (94–95%) than the p-methyl treatments alone (50% bloodfeeding inhibition and 51–70% personal protection) (Table 4). Mortality with the LLIN alone was 60% (Figure 2). Mortality was 100% when p-methyl DL and NWH where used whether alone or in combination with LLINs.
3. **Second combined intervention trial:** The results are presented in Table 5 and Figure 3. A total of 490 *An gambiae* ss were collected from the experimental huts during this trial (Table 5). Combining LLINs with pyrethroid DL did not show any improvement in mortality (48%) compared to the LLIN alone (44%) and pyrethroid DL alone (40%) (P>0.1) (Figure 3). Mortality was much higher (95%) when p-methyl DL was combined with LLINs.

**Table 3:** Personal protection and killing effect of p-methyl DL and NWH against pyrethroid resistant *An gambiae* in Valley du Kou, Burkina Faso (single intervention trial).

	Control (untreated DL on walls)	pyrethroid treated on walls DL	p-methyl DL on walls	p-methyl NWH on walls	p-methyl DL on walls and ceiling	p-methyl NWH on walls and ceiling
Total females caught	995^a^	464^b^	523^b^	841^a^	615^ab^	490^b^
Deterrence (%)	-	53	47	15	38	51
Total females blood fed	781	282	417	557	483	345
Blood feeding Inhibition (%)	-	20	4	19	7	15
**Personal Protection (%)**	**-**	**64**	**47**	**29**	**38**	**56**
Exiting rates (%)	18^a^	52^b^	36^c^	33^c^	40^c^	33^c^
Total dead	81	236	471	764	554	479
Corrected mortality	0^a^	38^b^	97^c^	97^c^	96^c^	99^c^
**Overall killing effect (%)**	**-**	**16**	**43**	**75**	**52**	**43**

Values along each row sharing the same letter superscript are not significantly different at the 5% level.

**Table 4:** Personal protection and killing effect of combining p-methyl DL and NWH with LLINs against pyrethroid resistant *An gambiae* in Valley du Kou, Burkina Faso (first combined intervention trial).

	Control (untreated net)	LLIN	p-methyl DL	p-methyl NWH	p-methyl DL+LLIN	p-methyl NWH+LLIN
Total females caught	81^a^	42^b^	68^ac^	45^bc^	38^b^	46^bc^
Deterrence (%)	-	48	16	44	53	43
Total females blood fed	64	11	28	18	3	4
Blood feeding Inhibition (%)	-	70	50	50	91	90
**Personal Protection (%)**	**-**	**83**	**56**	**72**	**95**	**94**
Total inside net (%)	36	0	-	-	0	0
Exiting rates (%)	33^a^	57^b^	53^b^	43^ab^	40^ab^	54^b^
Total dead	12	25	68	38	45	46
Corrected mortality	0^a^	54^b^	100^c^	100^c^	100^c^	100^c^
**Overall killing effect (%)**	**-**	**19**	**81**	**48**	**38**	**49**

Values along each row sharing the same letter superscript are not significantly different at the 5% level.

**Table 5:** Personal protection and killing effect of combining p-methyl DL vs pyrethroid DL with LLINs against pyrethroid resistant *An gambiae* in Valley du Kou, Burkina Faso (second combined intervention trial).

	Control	LLIN	pyrethroid DL+LLIN	p-methyl DL+LLIN
Total females caught	255^a^	72^bc^	67^b^	96^c^
Deterrence (%)	-	72	74	62
Total females blood fed	114	15	6	11
Blood feeding Inhibition (%)	-	53	80	74
**Personal protection (%)**	**-**	**87**	**95**	**90**
Total inside net (%)	29	1	0	2
Exiting rates (%)	31^a^	63^b^	61^b^	45^a^
Total dead	24	32	32	94
Corrected Mortality (%)	0^a^	39^b^	43^b^	98^c^
**Overall killing effect (%)**	**-**	**4**	**4**	**32**

Values along each row sharing the same letter superscript are not significantly different at the 5% level.

### Residual activity of insecticide

Mortality of laboratory reared susceptible *An gambiae* (Kisumu) tested in WHO cone bioassay on p-methyl DL and NWH, was 100% for the first 3–4 weeks of each of the trial but declined to 60–70% by the end of the trial. With pyrethroid DL, mortality remained 100% throughout the trial owing to the fact that the pyrethroid DL was factory coated using long-lasting technology.

### Selection of resistance alleles and genotypes

The *An gambiae* population was predominantly of the M-molecular form. Of 559 *An gambiae* samples which were randomly selected from weekly collections from the experimental huts during the trials, 98% were identified as belonging to the M-form of *An gambiae* ss.

A total of 732 and 656 *An gambiae* samples collected from the first two experimental hut trials were analysed for *kdr* and *ace 1^R^* respectively. The summary results on allele frequencies in live and dead collections are presented in Table 6. Genotype survival rates are presented in Table 7. The overall *kdr* allele frequency was 0.95 (n = 535) in the first trial and 0.86 (n = 197) in the second trial while the overall *ace-1^R^* allele frequency was 0.01 (n = 429) in the first trial and 0.03 (n = 228) in the second trial. There was no difference in the frequency of *kdr* alleles between live and dead collections from any of the treatments (P>0.05) (Tables 6). Analysis of genotype frequency (Table 7) showed that survival of the *kdr* heterozygotes (47% was no different from that of *kdr* homogygotes for resistance (52%) in the presence of LLIN (1^st^ trial: P = 0.71, 2^nd^ trial: P = 0.54).

**Table 6:** Comparative *kdr* and *ace 1^R^* allele frequencies in live and dead *An gambiae* ss collected from the experimental huts trials.

		*Kdr* allele freq (n)			*ace 1^R^* allele freq (n)		
Treatments		Live	Dead	*P*	Live	Dead	*P*
**S** ***ingle intervention trial***							
**1**	Control (untreated DL)	0.95 (140)	–	–	0.01 (97)	–	–
**2**	Pyrethroid DL	0.91 (51)	0.90 (51)	1	–	–	–
**3**	P-methyl DL (walls only)	1.0 (9)	0.97 (58)	1	0.05 (21)	0.00 (28)	0.18
**4**	P-methyl NWH (walls only)	1.0 (18)	0.95 (58)	0.34	0.00 (36)	0.00 (60)	1
**5**	P-methyl DL (walls and ceiling)	1.0 (18)	0.94 (61)	0.35	0.07 (28)	0.00 (64)	0.01
**6**	P-methyl NWH (walls and ceiling)	1.0 (3)	0.96 (68)	1	0.05 (29)	0.00 (66)	0.03
***Combined intervention trial***							
**1**	Control (untreated DL)	0.87 (63)	–	–	0.04 (81)	–	–
**2**	LLIN	0.83 (9)	0.85 (27)	1	–	–	–
**3**	P-methyl DL*	–	0.82 (31)	–	–	0.02 (34)	–
**4**	P-methyl NWH*	–	0.84 (19)	–	–	0.02 (31)	–
**5**	P-methyl DL+LLIN*	–	0.87 (23)	–	–	0.00 (42)	–
**6**	P-methyl NWH+LLIN*	–	0.94 (25)	–	–	0.07 (40)	–

no live mosquitoes were collected from huts with these treatments.

**Table 7:** Genotype selection by the single and combination treatments: percentage survival of *An gambiae kdr* and *ace 1^R^* genotypes collected from the experimental huts.

		*kdr*: % alive (live/total)			*ace-1^R^*: % alive (live/total)		
Treatments		SS	RS	RR	SS	RS	RR
**S** ***ingle intervention trial***							
**1**	Control (untreated DL)	-	100 (11/11)	100 (129/129)	100(96/96)	100(1/1)	-
**2**	Pyrethroid DL	-	47 (7/15)	52 (44/85)	-	-	-
**3**	P-methyl DL (walls only)	-	0 (0/4)	14 (9/63)	40 (19/47)	100 (2/2)	-
**4**	P-methyl NWH (walls only)	-	0 (0/6)	25 (18/70)	37 (36/96)	-	-
**5**	P-methyl DL (walls and ceiling)	-	0 (0/7)	22 (18/82)	27 (24/88)	100 (4/4)	-
**6**	P-methyl NWH (walls and ceiling)	-	0 (0/6)	5 (3/62)	28 (26/92)	100 (3/3)	-
***Combined intervention trial***							
**1**	Control (untreated DL)	100 (2/2)	100 (13/13)	100 (48/48)	100(75/75)	100(6/6)	-
**2**	LLIN	0(0/1)	33 (3/9)	23 (6/26)	-	-	-
**3**	P-methyl DL*	0 (0/3)	0 (0/5)	0 (0/23)	0 (0/33)	0 (0/1)	-
**4**	P-methyl NWH*	0 (0/2)	0 (0/2)	0 (0/15)	0 (0/30)	0 (0/1)	-
**5**	P-methyl DL+LLIN*	0 (0/1)	0 (0/4)	0 (0/18)	0 (0/42)	-	-
**6**	P-methyl NWH+LLIN*	-	0 (0/3)	0 (0/22)	0 (0/35)	0 (0/5)	-

no live mosquitoes were collected from huts with these treatments, SS = Homozygous susceptible, RS = Heterozygous, RR = Homozygous resistant.

While the *ace-1^R^* was low, there was generally a greater tendency for mosquitoes bearing the *ace-1^R^* allele to survive in huts with the p-methyl treatments alone in the single intervention trial. The *ace-1^R^* allele frequency was significantly higher in mosquitoes which survived in huts in which p-methyl treated DL and NWH were applied alone on walls and ceiling (P≤0.03) (Table 6). Analysis of genotype frequency showed that 100% (9/9) of *ace-1^R^* heterozygotes survived the p-methyl treatments but only 32% (105/323) of *ace-1* susceptibles survived (P = 0.001), indicating strong selection for the *ace-1^R^* resistance with the p-methyl interventions. In the second trial, all mosquitoes which entered the huts with p-methyl treatments whether applied alone or in combination with LLINs were killed (100% mortality). It was thus not possible to clearly demonstrate whether the combination prevents selection of the *ace-1^R^* gene compared to the single intervention of p-methyl (Tables 6 and 7). The low survival of *kdr* with the combination indicates that the p-methyl component might prevent the further selection of *kdr* resistance (Table 7).

## Discussion

In the current study, p-methyl treated DL and NWH outperformed pyrethroid treated DL by killing almost all malaria vectors which entered the huts. This superior performance was due to the fact that the vector population was very resistant to pyrethroids but susceptible to organophosphates. As pyrethroid resistance continues to spread, the use of non-pyrethroids like organophosphates and carbamates for IRS is increasing. With the exception of the newly developed micro-encapsulated formulation of p-methyl which lasts up to 9 months on cement walls [10], most organophosphate and carbamate insecticides though very toxic to mosquitoes are unfortunately short-lived when applied as IRS (2–4 months) compared to pyrethroids (up to 6 months) [17]. The development of long-lasting versions of p-methyl DL and NWH with residual activity over a number of years could significantly improve the usefulness of organophosphates in malaria vector control and enhance capacity to interrupt malaria transmission.

Increasing the level of wall coverage with p-methyl DL and NWH from walls only to walls plus ceiling did not have a major effect on the performance of these treatments in the experimental huts. Similar findings have been previously reported with pyrethroid DL [7]. This has positive implications for the scalability of these interventions since covering only walls as opposed to covering walls and ceilings is likely to be easier owing to the additional costs and practical difficulty of having to cover ceilings too. Pyrethroid DL was however found to induce significantly lower mortality when applied to two walls (20%) compared to all four walls (45%) [7]. It will be useful to investigate the performance of p-methyl DL and NWH when lower levels of wall coverage are achieved.

LLINs are capable of inducing high levels of mortality and providing significant personal protection to the user against a fully susceptible vector population. However, when faced with pyrethroid resistance, the insecticidal efficacy of the LLIN is significantly reduced, and the strength of the intervention may be compromised [8]. Nevertheless, with limited holes, LLINs may still provide partial protection against pyrethroid resistant vectors as shown in this study partly due to the physical barrier of the bed net and partly to the repellent property of the pyrethroid in the LLIN, and are thus much better than untreated nets or no nets at all. The current study demonstrates that the combining of p-methyl DL and NWH with LLINs induced high levels of mortality in a pyrethroid resistant population of malaria vectors and thus should restore transmission control to levels which cannot be achieved by the LLIN alone due to pyrethroid resistance. Mosquitoes would normally enter the room and feed on the sleeper before landing on the walls where they pick up the insecticide. The combination therefore showed potential to control transmission, largely due to the p-methyl DL and NWH components, and provide personal protection mainly due to the LLIN component. As with most IRS and IRS-like treatments, significant personal protection cannot be expected with p-methyl DL and NWH alone if only individual households are lined. However, if entire villages are covered, community protection should arise from the control of mosquito populations as occurs with IRS campaigns.

In contrast to p-methyl DL, combining pyrethroid DL and LLIN in the same hut did not show any improvement in mortality when compared to the LLIN alone. This can be attributed to the high level of pyrethroid resistance in the vector population and served as a positive control to demonstrate the importance of a non-resisted insecticide in the durable lining or NWH intervention. The present study confirms the fact that combining pyrethroid DL with pyrethroid LLIN for improved control of malaria transmission by a vector population which is resistant to pyrethroids may be a futile attempt and might not warrant the resources invested. Theoretical models suggest that the increased repellency posed by the additional pyrethroid wall treatment in the combination hut may also have decreased the chances of insect contact with insecticide [27]. The combining of pyrethroid IRS or IRS-like treatments with pyrethroid LLINs is generally not encouraged mostly because it exposes local vector populations to more intense selection pressure for pyrethroid resistance genes [9]. However some vector control programmes may continue to deploy pyrethroid IRS together with LLINs in the hope of improving transmission control. The performance of such a combination is likely to diminish if pyrethroid resistance exists in the targeted vector population and the threat of stronger resistance developing is more probable.

The frequency of the *kdr* (L1014F) mutation in *An gambiae* in Vallee du Kou 5 as observed in the current study was very high (0.89) and had increased remarkably from 0.28 in 2005 [28]. This confirms the rapid spread of the *kdr* among *An gambiae* populations across sub-Saharan Africa. Population genetic models suggest that the benefits of insecticide resistance management can be best achieved while resistance is still rare compared to when it is well established [20] [29] [30]. The high *kdr* allele frequency in the vector population could not permit a robust investigation into selection for *kdr* with the treatments tested. Nevertheless there was some evidence that selection of heterozygotes for *kdr* was no greater than selection of homozygotes for *kdr* and that selection of both genotypes would be delayed by the addition of p-methyl to an existing LLIN intervention. Meanwhile, mosquitoes bearing the *ace-1^R^* mutation were more likely to survive in huts when p-methyl DL and NWH were applied on walls and ceilings and no LLIN was in use. Because no live mosquitoes were collected from huts in the trial where p-methyl DL and NWH applied alone were compared with the combination of p-methyl DL/NWH and LLINs, it was not possible to demonstrate unequivocally the selective advantage or neutrality of resistance genes in the combination. But on the other hand there was similarly no evidence to indicate that any of the resistance alleles would be differentially selected by the combination, which is fair argument for applying the combination. There could also have been metabolic mechanisms of insecticide resistance in the vector population which in addition to the *kdr* may have contributed to the levels of phenotypic resistance to pyrethroids that was observed. Unfortunately, the absence of reliable DNA markers for the collection genes than can be up-regulated in metabolic resistance could not permit a realistic investigation into their selection in the current study. Apart from the resistance management potential, the study clearly shows that the combination would be a better option for controlling and providing protection against a vector population which is mostly resistant to pyrethroids but mostly susceptible to organophosphates than the single treatments alone. Considering the increasing reports of organophosphate resistance in malaria vectors in West Africa [31]–[33], there is opportunity to monitor what happens when the combination is deployed against a vector population which is partially resistant to both insecticides.

Residual activity with p-methyl treated DL and NWH declined over the course of the six weeks trials. This decline was faster than expected given the slow-release micro-encapsulated formulation of the insecticide used. The insecticide particles may have flaked off the treated materials during the course of the study. The study was designed as a proof of concept and the observed effect of p-methyl DL and NWH on mortality during these short term trials showed that mosquitoes will readily rest on p-methyl treated plastic wall linings and net wall hangings and be killed in the process. To maximise the benefits of these tools over IRS, the final product will need to have a residual activity that lasts for years rather than months. Advanced binding or incorporation technology needs to be developed to enable the development of a long lasting version of these tools.

Net wall hangings probably due to their light weight were much easier to hang on the walls than fixing of DL. Thus net wall hangings are potentially a more practical means of delivering insecticides indoors. Netting material is cheap and widely available. Treated NWH can be readily used in homes where IRS is short lived on mud walls. Treated wall netting can also be used to cover eave gaps as to reduce mosquito entry into the home. Small scale randomised trials are desirable to further assess the efficacy, acceptability and practicability of treated NWH in homes.

### Conclusion

Pirimiphos methyl treated DL and NWH show potential to provide improved control of pyrethroid resistant malaria vectors compared to currently available pyrethroid DL or IRS. Combining p-methyl DL/NWH with LLINs provides transmission control due mainly to the p-methyl DL/NWH component and personal protection due mainly to the LLIN component. Community wide protection and epidemiological impact are expected if p-methyl DL/NWH are deployed in combination with LLINs against vector populations which are partly or mostly resistant to pyrethroids but mostly susceptible to organophosphates. There was clear evidence from the hut trial that the single intervention would select for resistance to *kdr* and *ace-1^R^* and some evidence that the combination intervention would not select so strongly for resistance. NWH are a practical means of delivering insecticides indoors and need to be further explored. Advanced binding or incorporation technology is required to develop genuine long-lasting p-methyl DL or NWH and produce benefits over IRS.
